# Fabrication and Characterization of an Enzyme-Triggered, Therapeutic-Releasing Hydrogel Bandage Contact Lens Material

**DOI:** 10.3390/pharmaceutics16010026

**Published:** 2023-12-24

**Authors:** Susmita Bose, Chau-Minh Phan, Muhammad Rizwan, John Waylon Tse, Evelyn Yim, Lyndon Jones

**Affiliations:** 1 Centre for Ocular Research & Education (CORE), School of Optometry and Vision Science, University of Waterloo, 200 University Avenue West, Waterloo, ON N2L 3G1, Canada; s3bose@uwaterloo.ca (S.B.); lyndon.jones@uwaterloo.ca (L.J.); 2 Centre for Eye and Vision Research (CEVR), 17W Hong Kong Science Park, Hong Kong, China; 3 Chemical Engineering, University of Waterloo, 200 University Avenue West, Waterloo, ON N2L 3G1, Canada; muh.rizwan@utoronto.ca (M.R.); tse.jwt@gmail.com (J.W.T.); eyim@uwaterloo.ca (E.Y.)

**Keywords:** bandage contact lens, corneal wounding, gelatin methacrylate, GelMA+, MMP-9

## Abstract

Purpose: The purpose of this study was to develop an enzyme-triggered, therapeutic-releasing bandage contact lens material using a unique gelatin methacrylate formulation (GelMA+). Methods: Two GelMA+ formulations, 20% *w*/*v*, and 30% *w*/*v* concentrations, were prepared through UV polymerization. The physical properties of the material, including porosity, tensile strain, and swelling ratio, were characterized. The enzymatic degradation of the material was assessed in the presence of matrix metalloproteinase-9 (MMP-9) at concentrations ranging from 0 to 300 µg/mL. Cell viability, cell growth, and cytotoxicity on the GelMA+ gels were evaluated using the AlamarBlue^TM^ assay and the LIVE/DEAD^TM^ Viability/Cytotoxicity kit staining with immortalized human corneal epithelial cells over 5 days. For drug release analysis, the 30% *w*/*v* gels were loaded with 3 µg of bovine lactoferrin (BLF) as a model drug, and its release was examined over 5 days under various MMP-9 concentrations. Results: The 30% *w*/*v* GelMA+ demonstrated higher crosslinking density, increased tensile strength, smaller pore size, and lower swelling ratio (*p* < 0.05). In contrast, the 20% *w*/*v* GelMA+ degraded at a significantly faster rate (*p* < 0.001), reaching almost complete degradation within 48 h in the presence of 300 µg/mL of MMP-9. No signs of cytotoxic effects were observed in the live/dead staining assay for either concentration after 5 days. However, the 30% *w*/*v* GelMA+ exhibited significantly higher cell viability (*p* < 0.05). The 30% *w*/*v* GelMA+ demonstrated sustained release of the BLF over 5 days. The release rate of BLF increased significantly with higher concentrations of MMP-9 (*p* < 0.001), corresponding to the degradation rate of the gels. Discussion: The release of BLF from GelMA+ gels was driven by a combination of diffusion and degradation of the material by MMP-9 enzymes. This work demonstrated that a GelMA+-based material that releases a therapeutic agent can be triggered by enzymes found in the tear fluid.

## 1. Introduction

Corneal injury and subsequent damage to the corneal epithelium can lead to corneal scarring, vision loss, and potentially blindness. An estimated 1.5 to 2.0 million cases of monocular blindness are caused by ocular trauma and corneal ulceration annually [1]. Historically, the standard treatment for a corneal abrasion is the insertion of a lubricant onto the ocular surface, followed by patching the eye to prevent blinking, permitting the epithelium to heal under the patch. However, the use of an eye patch leads to frustration for the patient due to the loss of binocular vision. Furthermore, if the eye patch is not worn appropriately, then it can lead to delays in epithelial recovery, and it is also cumbersome for clinicians to assess the wound healing progress as this requires the removal of the patch [2,3]. Since the advent of more oxygen-transmissible silicone hydrogel materials in the late 1990’s, the standard of care for managing a corneal abrasion has switched to the use of bandage contact lenses (BCLs), in which a soft lens is typically worn for 7–10 days without removal [4,5,6,7].

The use of BCLs overcomes the aforementioned problems by allowing the patient to retain binocular vision while undergoing treatment, and clinicians can easily track the wound healing progression without having to remove the lens, as they can view the eye through the transparent lens material [2,3,8,9,10]. Unfortunately, current BCLs alone do not outperform ocular lubricants in terms of efficacy or speed of recovery, as they lack the ocular surface factors or therapeutics that are essential to aid ocular surface repair. Therefore, it would be beneficial if BCLs could also deliver topical therapeutic agents concurrently to the surface of the eye while in situ [11]. A soft BCL consists of hydrophilic polymers that can absorb large volumes of fluid [12,13]. As a result, these materials can also absorb and release soluble compounds, such as drugs, from their gel matrix [14]. However, previous studies have shown that commercial contact lens materials are unable to maintain sustained drug release, and the vast majority of adsorbed drugs are released within the first few hours of exposure to the eye, thus not providing a desirable release profile [15,16,17,18,19]. Modifications to current materials are needed to improve the release kinetics of currently available BCLs.

Among the various types of hydrogels available, gelatin is one of the most common polymers used in biomedical applications [20]. It is an amphoteric protein [20,21]. derived from the hydrolysis of collagen [22], a naturally occurring polymer in the human cornea [23]. It is a water-soluble, non-cytotoxic polymer with low immunogenicity, and is biodegradable and highly biocompatible [24,25,26]. Additionally, it contains many bioactive sequences, such as arginine-glycine-aspartic acid, which can facilitate cell attachment and adhesion [20,27,28], making it an ideal material for developing devices used in corneal wound healing [27]. Not surprisingly, gelatin-based hydrogels have been widely used in drug delivery and tissue engineering applications [29,30,31]. However, unmodified gelatin is relatively weak mechanically, making it a poor material for use as a BCL. These mechanical disadvantages of gelatin-based hydrogels can be overcome by chemical modifications or by integrating them with other monomers or polymers [20,32,33]. Gelatin methacrylate (GelMA), a derivative of porcine-derived gelatin, is produced by substituting the free amine groups of gelatin with methacrylate anhydride [20]. This polymer can be photo-crosslinked with a photoinitiator to produce a stronger permanent gel on exposure to ultraviolet (UV) radiation [20].

A previous publication showed that GelMA can be converted to GelMA+ by forming a gel at 4 °C before the UV crosslinking step [34]. The resulting gel has eight times higher mechanical strength than that of conventional GelMA [34]. The gelation step leads to the construction of triple helix and physical networks, which enhances the crosslinking density and produces a more homogenous microstructure [34].

An important feature of GelMA and its derivatives is the presence of matrix metalloproteinase-sensitive sites, which allows the gel to be biodegraded by matrix metalloproteinase (MMP) enzymes [35,36,37]. Several in vitro studies have shown that GelMA can be degraded in the presence of MMPs [36,37]. Of note with respect to the use of GelMA in the ocular environment is that elevated levels of MMP enzymes are observed following a corneal wound, in particular MMP-2 (72 kDa type IV collagenase) and MMP-9 (92 kDa type IV collagenase) [38,39,40,41]. Therefore, it would be possible to use GelMA as a primary polymer in an enzyme-triggered drug delivery system in which the release trigger is exposure to an MMP enzyme. The GelMA can be used to entrap drugs, drug nanoparticles, or therapeutics, which are then released when the gel is degraded by the MMPs present at the wound site. This study aimed to evaluate the release of a wound-healing therapeutic, bovine lactoferrin (BLF), from GelMA+ materials in the presence of MMP-9.

## 2. Materials and Methods

### 2.1. Materials

Gelatin Type A, BLF (80 kDa), methacrylic anhydride, and Irgacure 2959 were obtained from Sigma–Aldrich (St. Louis, MO, USA). MMP-9 was obtained from Gibco Thermo Fisher Scientific (Grand Island, NY, USA). The BLF ELISA kit was obtained from Bethyl Laboratories Inc. (Montgomery, TX, USA). The Spectrum^TM^ Spectra/Por^TM^ 4 RC Dialysis Membrane Tubing 12,000 to 14,000 Dalton MWCO was purchased from Fisher Scientific (Carlsbad, CA, USA). The EpiGRO^TM^ Human Ocular Epithelia Complete Media kit was obtained from Millipore Sigma (Burlington, MA, USA). The LIVE/DEAD^TM^ Viability/Cytotoxicity Kit for mammalian cells and the AlamarBlue^TM^ Cell Viability Reagent were purchased from Invitrogen^TM^ by Thermo Fisher Scientific (Eugene, OR, USA).

### 2.2. Gelatin Methacrylate Synthesis

The method for the synthesis of GelMA+ has been previously described [34]. In brief, 5 g of gelatin (type A) was dissolved in 50 mL of phosphate-buffered saline 1× (PBS) (10% *w*/*v*) with continuous magnetic stirring at 50–60 °C until the gelatin dissolved. 10 mL of methacrylic anhydride (20% *v*/*v*) was then added dropwise at 50–60 °C with continuous magnetic stirring and the reaction continued for 1 h. The resulting mixture was diluted with PBS and dialyzed in deionized (DI) water for 5 days at 40 °C using 12–14 kDa cut-off dialysis membrane tubes. The GelMA solution was then frozen at −80 °C and lyophilized.

### 2.3. Preparation of GelMA+ Hydrogels and BLF-Loaded GelMA+ Hydrogels

**Figure 1:**
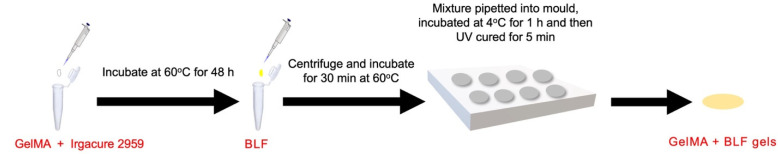
Preparation schematic for BLF-loaded GelMa+ hydrogels.

Lyophilized GelMA was mixed in a 1× PBS solution containing the photo-initiator 0.5% *w*/*v* Irgacure 2959 to obtain mixtures with 20% and 30% *w*/*v* of GelMA. The mixture was incubated at 60 °C for 48 h and centrifuged for 10 min at 5000 rpm. The mixtures were further incubated for 30 min at 60 °C before being carefully pipetted into an acrylic mould to create circular disks (thickness~0.65 mm, diameter~6 mm). The samples were then incubated at 4 °C for 1 h, before being exposed to UV radiation (360–420 nm) at an intensity of 32 mW/cm^2^ and polymerized in a Dymax ultraviolet curing chamber (Torrington, CT, USA) for 5 min to create GelMA+ gels. For the BLF-loaded GelMA+ hydrogels, 60 μL of 50 μg/mL of BLF was added to the mixture after the 48 h incubation period at 60 °C, then centrifuged for 10 min at 5000 rpm. Afterwards, the same procedure for the preparation of the GelMA+ hydrogel was followed (see Figure 1).

### 2.4. Physical Characterization

#### 2.4.1. Scanning Electron Microscopy

The pore size and surface morphology of the various gels were observed using an environmental scanning electron microscope (ESEM-FEI QUANTA^TM^ 250) manufactured by Field Electron and Ion Company (Hillsboro, OR, USA). The gels were kept in 1× PBS at room temperature (22–24 °C) to ensure complete swelling and to enable a clear picture of the morphology of the GelMA+ gels. The samples were observed under an accelerating voltage of 20 kV, in a low vacuum mode with a chamber pressure of 0.8 mbar. The electron beam energy was 20 keV. The sample surface was imaged with two detectors simultaneously: a large field detector to detect secondary electrons, which is more morphology sensitive, and a backscattering electron detector to detect backscattered electrons, which is more concentration sensitive.

#### 2.4.2. Enzymatic Degradation of the GelMA+ Hydrogels

The degradation of the 20% *w*/*v* and 30% *w*/*v* GelMA+ hydrogels was studied in the presence of varying concentrations of MMP-9. The MMP-9 concentrations were 0, 10, 50, 100, 300 (µg/mL). The circular disk-shaped GelMA+ samples were weighed to determine their initial weight (*W*_0_) and then placed in 2 mL of varying MMP-9 concentration solutions in a 24-well plate at 37 °C. The GelMA+ gels were then reweighed at predetermined time intervals (0 h, 4 h, 8 h, 12 h, 24 h, 48 h, 96 h, 144 h) to determine weight changes over time (*W_t_*). Before weighing, the gels were gently blotted using lens paper to remove any excess moisture. The MMP-9 solutions were replaced every day to maintain enzymatic activity. The percentage degradation was calculated using Equation (1).

(1) $$\mathrm{Percent}\;\mathrm{Degradation}=\frac{W_{0}-W_{t}}{W_{0}}\times 100\%$$

#### 2.4.3. Swelling Percentage and Water Content of the GelMA+ Hydrogels

To determine their swelling properties, the GelMA+ samples were incubated in 2 mL of PBS at 37 °C for 24 h. After 24 h, the samples were blotted dry using lens paper and weighed (*W_s_*). The same samples were then freeze-dried and weighed (*W_d_*). The percent swelling was calculated using Equation (2). The water content of the gels was measured similarly. Water content was calculated using Equation (3).

(2) $$\mathrm{Percent}\;\mathrm{Swelling}=\frac{W_{s}-W_{d}}{W_{d}}\times 100\%$$

(3) $$\mathrm{Water}\;\mathrm{Content}=\frac{W_{s}-W_{d}}{W_{s}}\times 100\%$$

#### 2.4.4. Mechanical Properties of the GelMA+ Hydrogels

The stiffness of the GelMA+ hydrogels was assessed using a Mandel–Shimadzu (AGS-X) tensile testing unit (Shimadzu Corp., Kyoto, Japan) at room temperature (22 °C to 24 °C). The GelMA+ samples were moulded in a rectangular shape of 7 cm by 1 cm by 0.7 cm (length × width × thickness) and then soaked in PBS for 24 h at room temperature. The samples were clamped with two steel clamps 5 mm apart, with Kim wipe tissues used to hold the edges of the gels to prevent the gels from breaking at the edge of the clamp and to prevent slippage. The rectangular-shaped gels were stretched at a rate of 1 mm/min to the breaking point with a load of 500 N. Young’s modulus was calculated from the slope of the linear region of the stress-strain curve.

#### 2.4.5. Optical Transmittance of the GelMA+ Hydrogels

The optical transmittance of gels with and without BLF was measured via a UV spectrophotometer (Biotek Citation 5; Winooski, VT, USA). The gels were placed in PBS in a 48-well plate and the measurements were performed through a wavelength range of 450–700 nm.

#### 2.4.6. In Vitro Release of Bovine Lactoferrin (BLF)

The in vitro release of BLF from 30% *w*/*v* GelMA+ gels was undertaken in the presence of varying concentrations of MMP-9 (0; 100; 300 μg/mL) at 37 °C. The samples were washed in 2 mL of PBS for 1 h to remove any loosely bound BLF. At *t* = 0, 1, 12, 24, 48, 72, 96, and 120 h, the samples were analyzed using the BLF ELISA kit. In brief, 100 μL of the test sample and the standard were added to the 96-well ELISA plate. HRP (streptavidin-conjugated horseradish peroxidase) and TMB (3,3′,5,5′-tetramethylbenzidine) reagents from the kit were added to each well to produce a colorimetric reaction. The change in yellow colour, proportional to the lactoferrin present in the sample, was measured at 450 nm absorbance using a UV spectrophotometer (BioTek Cytation 5; Winooski, VT, USA).

### 2.5. Biological Characterizations

#### 2.5.1. Cell Culture

The human papilloma virus (HPV) immortalized human corneal epithelial (HCEC) cell line was obtained as frozen from the Centre for Ocular Research & Education (CORE), School of Optometry and Vision Science at the University of Waterloo. The cells were cultured in tissue culture treated Corning^®^ cell culture flasks (Millipore Sigma, MA, USA) with a canted neck plug seal cap and a surface area of 25 cm^2^. The nutrient media consisted of EpiGRO^TM^ Human Ocular Epithelia Complete Media along with supplements of L-Glutamine, Epifactor O, Epifactor P, Epinephrine, rh Insulin, Apo transferrin, and Hydrocortisone hemisuccinate (Millipore Sigma, MA, USA). The cells were seeded at a ratio of 1:2 and grown in an incubator at 37 °C and 5% carbon dioxide.

#### 2.5.2. Cell Culture in the Presence of GelMA+ Hydrogels

Once the HCEC cells reached 90% confluency, they were seeded on 48-well VWR Tissue Culture (VWR International, Radnor, PA, USA) treated plates at a cell density of 5 × 10^4^ cells/cm^2^. The cells were grown in the nutrient media, as described above. Freshly prepared and sterile (UV sterilized) GelMA+ hydrogels of both the 20% *w*/*v* and 30% *w*/*v* formulations were placed carefully onto the cells in each well and incubated at 37 °C and 5% carbon dioxide. The disc-shaped gels were washed with sterile PBS for 4 min inside the cell culture hood prior to exposure to the cells. The gels were washed four times in 5 mL of fresh sterile PBS for 1 min each time to ensure that any unreacted photo crosslinker was removed. On the 5th day, the cell growth on the hydrogels was evaluated.

#### 2.5.3. Cell Mortality Assay

The AlamarBlue^TM^ cell viability assay (Thermo Fischer Scientific, Eugene, OR, USA) was conducted after 1, 5, and 7 days of incubation of the GelMA+ hydrogels with the immortalized HCEC cells. Freshly prepared and sterile (UV sterilized) GelMA+ hydrogels of both the 20% *w*/*v* and 30% *w*/*v* formulations were placed carefully onto the cells in each well and incubated at 37 °C and 5% carbon dioxide. The disc-shaped gels were washed with sterile PBS for 4 min inside the cell culture hood prior to exposure to the cells. The gels were washed four times in 5 mL of fresh sterile PBS for 1 min each time to remove any unreacted photocrosslinker. At each time point, the cell culture media was removed and then 0.5 mL of 10% *v*/*v* of the AlamarBlue^TM^ cell viability reagent prepared with serum-free DMEM/F12 media was added to each well. The resulting solution was then incubated at 37 °C and 5% carbon dioxide for 4 h. 100 µL of the solution from each well was transferred to a new 96-well plate. The fluorescence was measured (excitation 540 nm, emission 590 nm) using the BioTek Citation 5 (BioTek, Winooski, VT, USA).

#### 2.5.4. Live/Dead Assay

The cells were cultured and incubated in the presence of the GelMA+ hydrogels as previously described for 5 days. The media was changed on alternate days. Freshly prepared and UV-sterilized GelMA+ hydrogels were placed carefully onto the cells in each well and incubated at 37 °C and 5% carbon dioxide. Before placement onto the cells, the disc-shaped gels were washed to remove any unreacted photocrosslinker as described. The LIVE/DEAD^TM^ Viability/Cytotoxicity kit (Thermo Fischer Scientific, Eugene, OR, USA) was used to stain the cells and the procedure was performed as described by the manufacturer. 20 µL of 2 mM of EthD-1 was added to 10 mL of sterile PBS, resulting in a 4 µM EthD-1 solution. 5 µL of 4 mM calcein AM stock solution to the 10 mL EthD1 solution. The solution was vortexed to ensure thorough mixing. The growth media was withdrawn from the wells of the 48-well plate containing the cells and the resulting approximately 2 µM Calcein AM and 4 µM EthD-1 solution was then added directly to cells containing the GelMA+ gels. The cells were incubated with dye at room temperature (22–24 °C) for 20–30 min. Images were obtained with Citation 5 (BioTek, Winooski, VT, USA) via fluorescence microscopy on the 5th day.

#### 2.5.5. Statistical Analysis

Statistical analysis and graphs were plotted using GraphPad Prism 6 software (GraphPad, La Jolla, CA, USA). An analysis of variance (ANOVA) and a post-hoc Tukey’s test were performed when necessary to determine the statistical significance between different conditions. A *p*-value of <0.05 was considered significant.

## 3. Results

### 3.1. Physical Characterization

#### 3.1.1. Scanning Electron Microscopy Images

**Figure 2:**
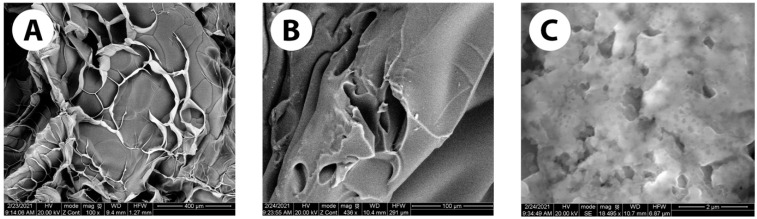
SEM images of (**A**) pre-polymerised GelMA (**B**) 20% *w*/*v* GelMA+ hydrogel and (**C**) 30% *w*/*v* GelMA+ hydrogel.

Figure 2 demonstrates the surface morphology and pore size of the GelMA+ hydrogels at concentrations of 20% *w*/*v* and 30% *w*/*v* via SEM. The images provide a visual representation of the internal porous structure of both GelMA+ formulations. In Figure 2A, the morphology of the pre-polymerized GelMA exhibits a highly porous surface, with estimated pore sizes ranging from 150 µm to 300 µm. Figure 2B,C exhibit the internal porous structures of the 20% and 30% *w*/*v* GelMA+ hydrogels, respectively. The surface of the 20% *w*/*v* GelMA+ exhibits a porous texture, characterized by pore sizes ranging from 30 µm to 90 µm. In contrast, the 30% *w*/*v* GelMA+ surface is more compact, with pore sizes measuring between 0.078 µm and 0.8 µm.

#### 3.1.2. Enzymatic Degradation of GelMA+ Hydrogel

**Figure 3:**
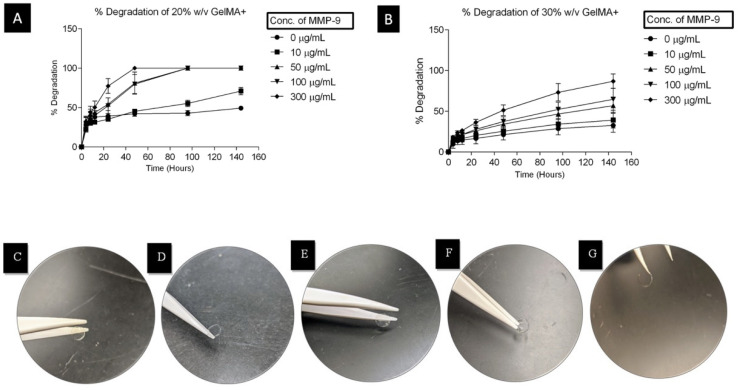
Degradation profile of (**A**) 20% *w*/*v* GelMA+ hydrogel in varying MMP-9 concentrations, (**B**) 30% *w*/*v* GelMA+ hydrogel in varying MMP-9 concentrations, (**C**) the shape of 20% *w*/*v* GelMA+ hydrogel in PBS after 48 h, (**D**) the shape of 20% *w*/*v* GelMA+ hydrogel in 100 µg/mL of MMP-9 after 48 h, (**E**) the shape of 30% *w*/*v* GelMA+ hydrogel in PBS after 144 h, (**F**) the shape of 30% *w*/*v* GelMA+ hydrogel in 100 µg/mL of MMP-9 after 144 h, (**G**) the shape of 30% *w*/*v* GelMA+ hydrogel in 300 µg/mL of MMP-9 after 144 h. The degradation was conducted at 37 °C.

Figure 3 shows the degradation of the GelMA hydrogels in MMP-9 over 144 h (6 days). Figure 3A,B shows the degradation profile of GelMA+ in the presence of different MMP-9 concentration solutions. For both formulations of GelMA+ gels, the degradation rate increased with increasing concentrations of MMP-9 (*p* < 0.0001). The 20% *w*/*v* GelMA+ gels degraded faster than the 30% *w*/*v* GelMA+ gels (*p* < 0.001). On the second day (48 h), the circular-shaped 20% *w*/*v* GelMA+ gels completely degraded in the presence of 300 µg/mL of MMP-9 without any gel remnants. In contrast, the 30% *w*/*v* GelMA+ gels took almost 144 h to degrade approximately 95% of their original weight, leaving behind only a thin piece of the original gel.

#### 3.1.3. Swelling Profile and Water Content of GelMA+ Hydrogel

Table 1 shows the swelling ratio and the water content of 20% *w*/*v* and 30% *w*/*v* GelMA+ hydrogels (*n* = 4). Both formulations of GelMA+ substantially were swelled over 24 h in the presence of PBS to ensure that an equilibrium was achieved. After 24 h, it was observed that the 20% *w*/*v* GelMA+ gels swelled more (*p* < 0.05) than the 30% *w*/*v* GelMA+. The water content of 20% *w*/*v* GelMA+ gels was significantly higher as compared to 30% *w*/*v* GelMA+ gels (*p* < 0.05).

#### 3.1.4. Tensile Test

Table 1 shows the influence of the increasing GelMA+ concentration on the tensile strain and Young’s Modulus (*n* = 3). The higher GelMA+ concentration increased the tensile strain values (*p* < 0.05). The 20% *w*/*v* GelMA+ gels were softer with a Young’s modulus value of 2.04 MPa. The 30% *w*/*v* GelMA+ gels were stiffer with Young’s modulus value of 2.80 MPa with reduced elongation at breakpoints.

#### 3.1.5. Optical Transmittance

**Figure 4:**
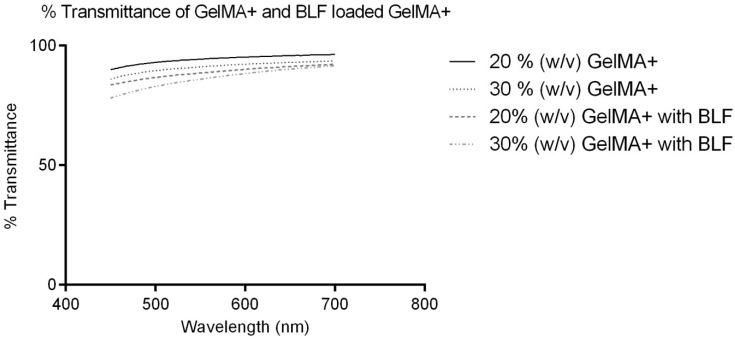
Optical transmittance of GelMA+ gels and BLF-loaded GelMA+ gels measured at a wavelength range of 450 nm to 700 nm.

The optical clarity (*n* = 5) of both the blank 20% *w*/*v* GelMA+ and 30% *w*/*v* GelMA+ gels and BLF-loaded GelMA+ gels (Figure 4) were measured between 450–700 nm using the Citation 5UV spectrophotometer. The transmittances of the 30% *w*/*v* GelMA+ decreased significantly (*p* < 0.0001) as opposed to 20% *w*/*v* GelMA+. The blank 20% *w*/*v* GelMA+ gels exhibited approximately 90% transmittance at 450 nm. At 630 nm, the transmittance of 20% *w*/*v* GelMA+ was 95.59 ± 1.80%. At 450 nm, the blank 30% *w*/*v* GelMA+ gels exhibited 86.07 ± 3.88% transmittance, and at 630 nm, the transmittance was 92.69 ± 2.96%. The 20% *w*/*v* GelMA+ gels loaded with BLF exhibited 83.61 ± 3.47% transmittance at 450 nm. At 630 nm, the transmittance of the same gel was 90.82 ± 3.06%. At 450 nm, the BLF-loaded 30% *w*/*v* GelMA+ gels exhibited 78.15 ± 2.64% transmittance, and at 630 nm, the transmittance was 89.49 ± 1.29%.

#### 3.1.6. In Vitro Release of Bovine Lactoferrin

**Figure 5:**
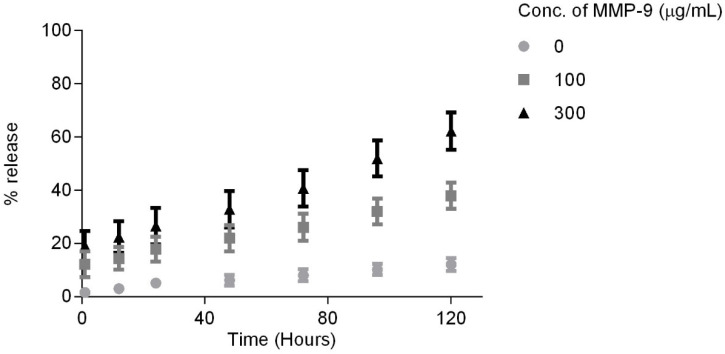
In vitro release of Bovine Lactoferrin from 30% *w*/*v* GelMA+ in the presence of varying MMP-9 concentrations at 37 °C.

The cumulative percent in vitro release kinetics of the BLF (*n* = 4) is shown in Figure 5. The release of BLF from the 30% *w*/*v* GelMA+ hydrogel matrix significantly increased with increasing concentration of MMP-9 (*p* < 0.0001). The amount of BLF released increased over time for all MMP-9 concentrations (*p* < 0.0001). Due to the initial washing of the gels for an hour, there was no burst release observed. The results show that the release of BLF from the gels was primarily driven by the enzyme present in the solution.

### 3.2. Biological Characterization

#### 3.2.1. Cell Growth on GelMA+ Gels

**Figure 6:**
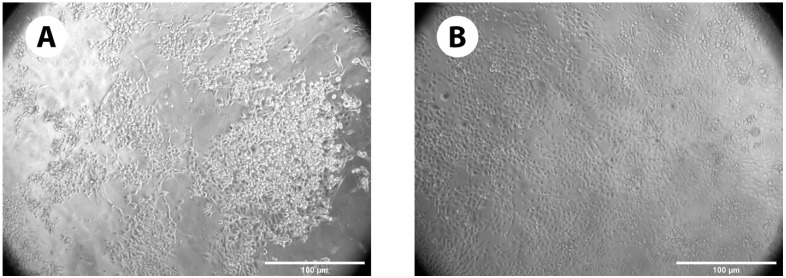
(**A**) Growth of immortalized HCEC cells on 20% *w*/*v* GelMA+ gels, (**B**) Growth of immortalized HCEC cells on 30% *w*/*v* GelMA+ gels. On the 5th day, the cell growth on the respective hydrogels was observed.

Figure 6 the growth and attachment of the immortalized HCEC cells in the presence of both the formulations of GelMA+ gels (*n* = 4). With 30% *w*/*v* GelMA+ gels, a greater amount of cell growth and attachment was observed as compared to 20% *w*/*v* GelMA+. The images were observed phase-contrast microscopy with a 20× objective. The images were captured on the 5th day using Zeiss AxioVision Software (White Plains, NY, USA).

#### 3.2.2. Cell Mortality Assay

**Figure 7:**
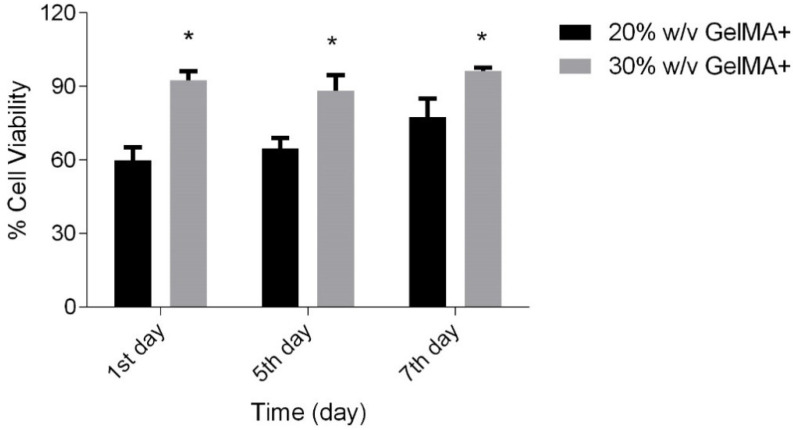
Percentage of HCEC cell viability in the presence of 20% *w*/*v* and 30% *w*/*v* GelMA+ respectively in comparison to control. * *p* < 0.05.

Figure 7 shows the percentage of cells viable on the GelMA+ hydrogels (*n* = 4) on the 1, 5, and 7 days of incubation as measured by the AlamarBlue^TM^ assay. The cell viability was compared to the control, where the cells were grown in the absence of the GelMA+ gels, only in the presence of EpiGrow media. On the 7th day, 20% *w*/*v* GelMA+ hydrogel showed 80% cell viability, whereas the 30% *w*/*v* GelMA+ showed almost 95% cell viability.

#### 3.2.3. Live/Dead Assay

**Figure 8:**
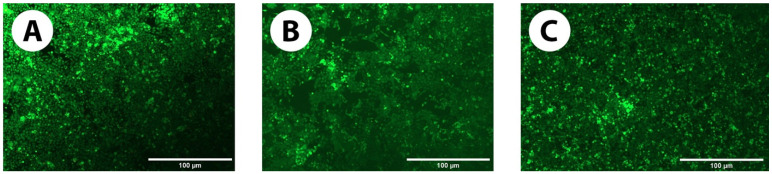
Live/Dead HCEC cells distribution on (**A**) Control (No GelMA+), (**B**) 20% *w*/*v* GelMA+, (**C**) 30% *w*/*v* GelMA+. The green-coloured (calcein AM) dots denote the presence of live cells and the red-coloured dots (EthD-1) denote the presence of dead cells. These images were captured after 5 days of incubation of the HCECs on the respective GelMA+. The images show the higher number of live cells in the presence of GelMA+ which is comparable to the control.

Figure 8A–C show the live and dead cell distribution after 5 days in the culture media control (no GelMA+), cells incubated with 20% *w*/*v* GelMA+ and cells incubated with 30% *w*/*v* GelMA+ (*n* = 4). The experiments were repeated on different days. Cells that were stained green (Calcein-AM) were live cells whereas the cells that were stained red (EthD-1) were dead cells. For both the formulations of GelMA+, a large number of cells remained alive with no signs of cytotoxicity (as evident from the green colour of Calcein-AM) when compared to the control after 5 days.

## 4. Discussion

GelMA hydrogels have been previously used in various biomedical devices [42,43,44,45], but the material lacks the mechanical strength required for high wear-tear applications, such as for use on the eye [34]. In a previous study, the modification of a conventional GelMA hydrogel using a sequential hybrid crosslinking process was described, involving both physical and chemical crosslinking, to improve the mechanical properties of the resulting material [34]. The new material, GelMA+, demonstrated an 8-fold increase in mechanical strength as compared to GelMA. The extra incubation period leads to improved triple helix and physical network formation leading to enhanced crosslinking. The resulting GelMA+ material has significantly improved mechanical strength and exhibits slower biodegradation kinetics (both in vitro and in vivo) than GelMA [34].

The physical characterization (porosity, tensile modulus, and water swelling) and biological parameters (cell viability and spreading) of GelMA hydrogels are important to determine the suitability of these hydrogel polymers for different biomedical applications. The SEM images (Figure 2) showed the porous nature of the GelMA+ hydrogel. The porosity of the hydrogel affects both the drug uptake and release [46,47]. Previous studies on GelMA have shown the porous nature of the hydrogel, with pore sizes ranging from 50 µm to 77 µm [48,49]. The pore size of the fully crosslinked GelMA+ was considerably less compared to the pre-polymerized GelMA. The pore size of pre-polymerized GelMA was around 150 µm to 300 µm, whereas the pore size for 20% and 30% *w*/*v* GelMA+ were around 30–90 µm and 0.078–0.8 µm respectively. With an increase in the concentration of the polymer, there was an increase in the crosslink density, which was also observed in previous studies [50,51]. The increase in cross-linking density concurrently leads to a decrease in the pore size.

Both the formulations showed a considerably high tensile strength. The 30% *w*/*v* GelMA+ was stiffer, with a tensile strain of 181.85 ± 25.25 kPa, as compared to the 20% *w*/*v* GelMA which had a tensile strain of 133.06 ± 8.98 kPa (see Table 1). The higher modulus can be attributed to an increase in crosslink density, which limits the material’s ability to deform. These high tensile values are important to maintain the original shape and physical dimensions of any contact lens or ocular drug-delivering insert made for exposure to the ocular surface, which would be exposed to blinking [52]. The 30% *w*/*v* GelMA+ was stiffer, with a Young’s modulus of 2.8 MPa, as compared to the 20% *w*/*v* GelMA, which had a tensile modulus of 2.0 MPa (Table 1). This shows that the mechanical property of the GelMA+ hydrogel could be effectively regulated by increasing the GelMA+ concentration. To prepare GelMA+ gels with tensile moduli values of commercial soft contact lens materials, which are typically around 0.3 to 0.6 MPa [53], further tuning of the GelMA+ material is needed.

Hydrogels consist of hydrophilic polymeric networks capable of imbibing large amounts of water [54,55]. A previous study demonstrated that hydrogels with smaller pore sizes have lower swelling ratios [56]. As expected (Table 1), the 20% *w*/*v* GelMA+, with its lower cross-link density and larger pore sizes, swelled more than the 30% *w*/*v* GelMA+. Crosslink density also affected the rate of degradation of the GelMA+ gels. In this study, the degradation was also shown to be dependent on the presence of collagenase enzymes such as MMPs, with increasing amounts of MMP-9 concentration increasing the degradation rate (Figure 3). The 20% *w*/*v* GelMA+ was completely degraded in the 300 µg/mL MMP-9 in 2 days, without any remnant of the original polymer. However, the 30% *w*/*v* GelMA+ took almost 6 days to degrade to 95% of the original polymer in the same MMP-9 concentration. The results suggest that increasing the GelMA+ cross-link density can be used to extend the degradation time for the GelMA+ hydrogels by MMP enzymes. Figure 3C–G shows the shape of the gels during the stages of degradation. In Figure 3B,D, it was observed that the edges of the circular gels remained after degradation. This can be attributed to the shape of the mould in which the gels were formed, which is thicker on the edges than the centre. Based on this study, the 30% *w*/*v* GelMA+ would be an ideal candidate for encapsulation of drugs and therapeutics.

The transmittance of contact lenses should be above 90% for optical clarity [57]. It is evident from Figure 4 that the transmittance of both blank GelMA+ formulations is above 90% in the visible light range. However, the 30% *w*/*v* GelMA+ gels exhibited lower (*p* < 0.0001) transmittance compared to 20% *w*/*v* GelMA+. This is likely due to the higher concentration of polymer in the 30% *w*/*v* GelMA+ [58]. With BLF-loaded GelMA+ gels, the transmittance values were lower than the blank gels (Figure 4) owing to the presence of BLF molecules in the GelMA+ matrix. Commercial soft contact lenses have a central thickness ranging from 0.06 mm to 0.24 mm [59]. The GelMA+ gels in this study have a thickness of around 0.65 mm. The transmittance value of both the blank and BLF-loaded GelMA+ formulations would increase if the gels were made thinner [60].

Several papers have shown that GelMA is a favourable biopolymer for cell growth [49,61,62,63,64] and hence proliferation and cytotoxicity tests were conducted to evaluate the biocompatibility of GelMA+. Figure 6A,B indicated that the HCEC thrived and proliferated in the presence of GelMA+, with greater cell growth on the 30% *w*/*v* GelMA+ hydrogel compared to 20% *w*/*v* GelMA+. It was hypothesized that at higher GelMA+ concentrations, there are more cell-attachment sites (Arg-Gly-Asp (RGD)). The greater spreading of GelMA+ may also be associated with the material’s increased stiffness, which is required as an adequate surface/matrix for HCEC growth [65]. In line with the Alamar Blue data observed in the cell growth on GelMA+ gels (Figure 7), more cells were viable in the presence of 30% *w*/*v* GelMA+ gels (*p* < 0.0001). indicating that the cells preferred the presence of GelMA+. The Live/Dead cell assay (Figure 8A–C) indicated that neither formulation was cytotoxic to the HCEC cells. A large number of cells were viable for a period of 5 days with very few dead cells, as indicated by the red dots (EthD-1) in the live/dead cell assay images. For both formulations of GelMA+, a considerably large number of cells remained alive with almost no signs of cytotoxicity. Overall, these results indicated that the immortalized HCEC could proliferate over 5 days on all GelMA+ hydrogels, with greater attachment and proliferation on 30% *w*/*v* GelMA+.

Based on the porosity of the GelMA+ gels, a high molecular weight compound would be an ideal candidate for entrapment and release from the gel when degraded by collagenase enzymes. It was hypothesized that smaller molecules could simply diffuse from the gels at a faster rate than the rate of degradation. For this study, BLF was used as a model therapeutic for wound healing, which has a molecular weight of around 80 kDA [66]. Lactoferrin, an iron-binding monomeric glycoprotein [67], is produced by the epithelial cells of different mammalian organs and found in several secretions, including milk, saliva, tears [68], digestive secretions, nasal secretions, colostrum, and vaginal fluids, with colostrum and milk producing the highest amount of lactoferrin. It can be obtained from several mammalian species [69,70,71]. BLF has been reported to promote wound healing [66,72,73,74] and has shown promising results in the closure of alkali-wounded corneal epithelial cells [66,72]. It has been shown that the C-lobe of BLF is primarily responsible for the healing of wounded corneal epithelial cells [75].

MMPs play an important role in wound healing and inflammation [76]. These enzymes are responsible for the cleaving and remodelling of epithelial basement components and tight junction proteins [77,78]. MMPs are proteolytic enzymes that are produced where ocular surfaces are stressed [79,80]. The presence of pro MMP-9 in the tears of healthy patients was found at around 20.32 ± 5.21 ng/mL. However, patients suffering from conjunctivochalasis had MMP-9 concentrations around 223.4 ± 74.53 ng/mL [81]. In another study, active MMP-9 among healthy patients was reported at 8.39 ± 4.70 ng/mL, while patients suffering from severe dysfunctional tear syndrome showed a very high MMP-9 activity of 381.24 ± 142.83 ng/mL [82]. The presence of MMP-9 on the ocular surface can be utilized to enhance the release of active therapeutic agents from GelMA+ materials, and subsequently aid in corneal wound healing. Here, as a proof of concept, a higher level of enzymatic concentration than that typically found on the ocular surface was chosen to trigger the release of BLF from 30% *w*/*v* GelMA+ gels to demonstrate the correlation between the enzymatic concentration and the release of the therapeutic agent. However, more work will be needed to design materials that exhibit the same degradation kinetics at physiological concentrations of MMP9s.

As the 30% *w*/*v* GelMA+ was found to be a favourable candidate for the wound healing material, it was chosen as the hydrogel polymer to study the triggered release of BLF dispersed in its matrix over a period 5 days in the presence of varying MMP-9 concentrations. The GelMA+ was able to release BLF over the specified study period in an increasing manner (Figure 5). The release kinetics of BLF from the gels did not show a burst release within the first hour, which is normally observed in drug release studies from hydrogels [15,18,19,83]. It was hypothesized that the initial wash step for an hour removed most of the loosely bound BLF on the surface or sub-surface of the gels, which was around 14% of the total therapeutic concentration, which could have contributed to a burst release. In the absence of MMP-9, only 12.06 ± 3.41% of the total BLF was released after 120 h, which was due to passive diffusion. The overall results showed that the release of the BLF is dependent on both diffusion and enzyme concentration but with a higher impact from the latter. Thus, it is possible to control the release of the drug therapeutic from the GelMA+ by adjusting the polymer and enzyme concentration. Depending on the enzymes available at the wounded site, the GelMA+ gels can be formulated similarly so that they degrade completely in situ, releasing the therapeutic or drug to assist in wound healing.

One advantage of an enzyme-degradable biomaterial is that it does need to be removed while it degrades in situ. It can be used for sustained and controlled drug delivery to deliver drugs to the site of action in the body [84,85]. As observed from the BLF release profile, the release of the therapeutic agent is directly proportional to the presence of MMP-9. This implies that the drug release will respond to the wound’s severity. Large wounds will have higher MMP levels [86,87] and this in turn will degrade the GelMA+ faster, leading to a higher release of the therapeutic agents. The main disadvantage of a GelMA+ bandage contact lens is that it would cause vision problems as it degrades. One alternative is to incorporate this material as a contact lens skirt or ring implant or formulate the material as an ocular insert that is inserted under the lower lid, where slow degradation would not impact vision but would release the therapeutic of interest.

## 5. Conclusions

This study investigated the use of GelMA+ gels at different concentrations (20% and 30% *w*/*v*) as potential materials for therapeutic bandage contact lenses or ocular inserts to treat recurrent corneal erosion or other ocular surface injuries. GelMA+ gels demonstrated degradation in the presence of MMP-9, an enzyme upregulated during corneal wounds. The release of BLF from the GelMA+ gel was facilitated by MMP-9. These findings suggest that GelMA+ gels hold promise as a biomaterial to promote corneal wound healing. Further research can explore ways to optimize the gel properties for faster degradation at lower, physiologically relevant MMP-9 concentrations.

## Figures and Tables

**Table 1:** Percent swelling, water content, tensile strain, and Young’s modulus of both GelMA+ formulations.

Formulation	20% GelMA+	30% GelMA+
**% Swelling**	303.69 ± 6.95	234.68 ± 8.98
**Water content (%)**	74.85 ± 0.67	70.85 ± 1.81
**Tensile strain (kPa)**	133.06 ± 8.98	181.85 ± 25.25
**Young modulus (MPa)**	2.04 ± 0.16	2.80 ± 0.74

## Data Availability

The data can be shared up on request.
